# Supplementary material for: Characterization of Endofungal Bacteria and Their Role in the Ectomycorrhizal Fungus Helvella bachu
Source: J Fungi (Basel). 2024 Dec 23;10(12):889. doi: 10.3390/jof10120889 (PMC11677703; doi:10.3390/jof10120889)
Supplement: Supplementary file 1 [file jof-10-00889-s001.zip › Supplementary Figures S1 to S2.pdf]

Supplementary materials

Manuscript title:

Characterization of Endofungal Bacteria and Their Role in the Ectomycorrhizal Fungus *Helvella bachu*

Caihong Wei[1,2,3], Mengqian Liu[1], Guoliang Meng[1], Miao Wang[1], Xin Zhou[1], Jianping Xu[4], Jianwei Hu[2,3], Lili Zhang[2,3*], Caihong Dong[1*]

[1] State Key Laboratory of Mycology, Institute of Microbiology, Chinese Academy of Sciences, Beijing 100101, China; liumengqian19@mails.ucas.ac.cn, menggl@im.ac.cn; mwang2136@gmail.com; zhouxin@im.ac.cn; dongch@im.ac.cn
[2] College of Life Science and Technology, Tarim University, Alar 843300, China 18399410069@163.com; skyhjw@taru.edu.cn; zhanglily@taru.edu.cn
[3] Xinjiang Production and Construction Corps Key Laboratory of Protection and Utilization of Biological Resources in Tarim Basin, Alar 843300, China
[4] Department of Biology and Institute of Infectious Diseases Research, McMaster University, Hamilton, Ontario,
*Correspondence: zhanglily@taru.edu.cn; dongch@im.ac.cn

Information:

1. Supplementary Figures S1 to S2.
2. Supplementary Tables S1 to S14.

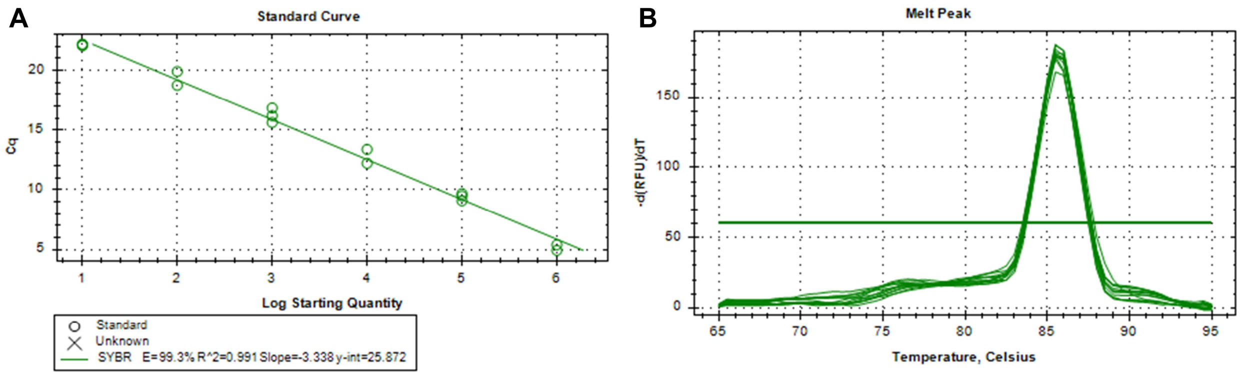

Figure S1. Real-time quantitative PCR based on the 16S rRNA gene. (A) Standard curve of the plasmid standard; (B) The melting curve

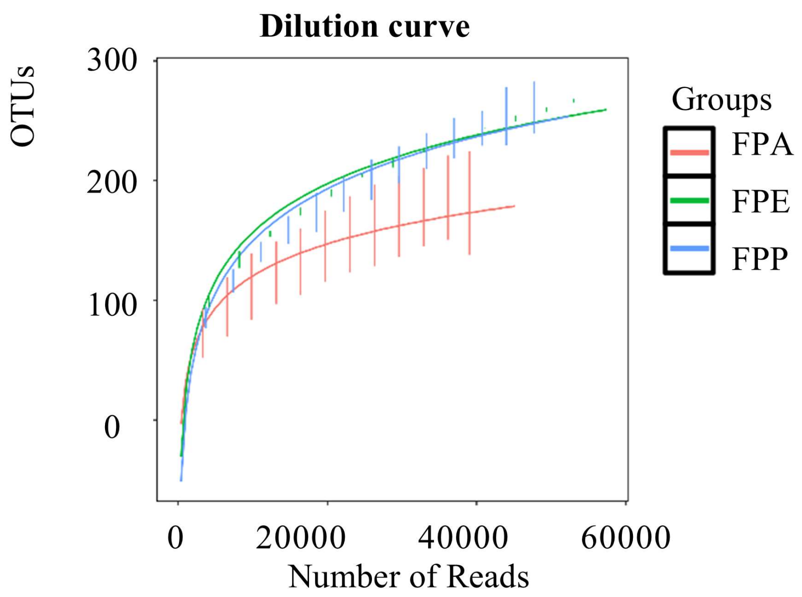

Figure S2. Dilution curve for the data of full-length 16S rRNA gene sequencing
