# Supplementary material for: Characterization of Endofungal Bacteria and Their Role in the Ectomycorrhizal Fungus Helvella bachu
Source: J Fungi (Basel). 2024 Dec 23;10(12):889. doi: 10.3390/jof10120889 (PMC11677703; doi:10.3390/jof10120889)
Supplement: Supplementary file 1 [file jof-10-00889-s001.zip › Supplementary Tables S1 to S14.pdf]

Table S1. Possible EFBs identified during genome sequencing analysis

N.	Genus name	reads
1	*Acidovorax*	22
2	*Pedobacter*	20
3	*Agrobacterium*	15
4	*Stenotrophomonas*	15
5	*Aminobacter*	11
6	*Chryseobacterium*	7
7	*Devosia*	7
8	*Ochrobactrum*	7
9	*Flavobacterium*	6
10	*Bosea*	4
11	*Legionella*	4
12	*Pseudomonas*	4
13	*Sphingopyxis*	4
14	*Ensifer*	2
15	*Rhizobium*	2
16	*Tardiphaga*	2
17	*Variovorax*	2
18	*Achromobacter*	1
19	*Bacteroidetes*	1
20	*Bradyrhizobium*	1
21	*Chitinophaga*	1
22	*Paenacidovorax*	1
23	*Pseudoxanthomonas*	1
24	*Rhodopseudomonas*	1
25	*seudomonas*	1
26	*Sinorhizobium*	1
27	*Gammaproteobacteria*	1
	total	144

Table S2. Data statistics of EFBs in *Helvella bachu* fruiting body associated with three hosts by full-length 16S rRNA gene sequencing

N.	Sample	input	Primer removed	Length filterd	Chimeras removed	Organelle removed	Percentage of pass filter
1	FPE* 1	85,507	85,507	57,674	50,921	50,709	0.593
2	FPE 2	92,037	92,037	62,476	57,656	57,428	0.624
3	FPE 3	89,882	89,882	60,743	55,500	55,323	0.6155
4	FPP* 1	79,534	79,534	53,699	52,796	52,320	0.6578
5	FPP 2	79,325	79,325	53,492	50,824	50,066	0.6312
6	FPP 3	67,975	67,975	45,737	43,451	42,359	0.6232
7	FPA* 1	69,198	69,198	47,154	45,406	45,157	0.6526
8	FPA 2	68,574	68,574	46,697	41,484	41,037	0.5984
9	FPA 3	67,341	67,341	45,467	37,147	36,454	0.5413

* FPE: *P. euphratica;* * FPP: *P. pruinosa;* * FPA: *P. alba* var. *Pyramidalis*.

Table S3. Statistical data of EFBs of *Helvella bachu* under different hosts

	**Phylum**	**Class**	**Order**	**Family**	**Genus**	**Species**
FPE 1	13	18	50	73	116	157
FPE 2	14	19	48	68	118	161
FPE 3	11	16	47	64	111	155
Populus euphratica	14	20	62	88	151	213
FPP 1	12	19	52	82	161	209
FPP 2	12	22	52	79	143	178
FPP 3	11	18	47	70	135	162
Populus pruinosa	14	28	70	108	211	281
FPA 1	7	10	30	49	84	110
FPA 2	10	15	40	61	119	151
FPA 3	8	10	37	56	104	136
Populus alba var. *pyramidalis*	11	18	52	84	156	209
Total	17	32	88	135	259	367

Table S4. Reads composition of the EFBs of *Helvella bachu* under different hosts at phylum level

	FPE		FPP		FPA	
	**average value**	**Percentage (%)**	**average value**	**Percentage (%)**	**average value**	**Percentage (%)**
Planctomycetota	5	0.01	5	0.01	1	0
Verrucomicrobiota	15	0.03	5	0.01	45	0.11
Patescibacteria	16	0.03	9	0.02	1	0
Proteobacteria	31335	57.51	40749	84.46	32388	79.22
Bacteroidota	22755	41.76	6900	14.3	8379	20.5
Acidobacteriota	3	0.01	15	0.03	3	0.01
Bdellovibrionota	342	0.63	1	0	0	0
Myxococcota	1	0	1	0	1	0
Actinobacteriota	5	0.01	50	0.1	13	0.03
Firmicutes	2	0	511	1.06	51	0.12
Armatimonadota	4	0.01	0	0	0	0
Chloroflexi	0	0	2	0	0	0
Dependentiae	2	0	0	0	0	0
Spirochaetota	1	0	0	0	0	0
Gemmatimonadota	0	0	1	0	0	0
Desulfobacterota	0	0	0	0	1	0
Total	54487	100	48248	100	40883	100

Table S5. Common genus of EFBs in *Helvella bachu* associated with different hosts

**Host plant**	**FPA**	**FPA**	**FPE**	**FPE**	**FPP**	**FPP**
**Genus**	**Average abundance**	**Relative abundance**	**Average abundance**	**Relative abundance**	**Average abundance**	**Relative abundance**
Acidovorax	1852.67	6.35%	2935	11.84%	11611	27.74%
Agrobacterium	7.33	0.03%	14	0.06%	3254.33	7.77%
Aminobacter	3.33	0.01%	364.33	1.47%	59.67	0.14%
Bosea	107.67	0.37%	2277.33	9.19%	80.33	0.19%
Bradyrhizobium	7	0.02%	38.67	0.16%	4	0.01%
Brevundimonas	1520.33	5.21%	7.33	0.03%	9.33	0.02%
Chryseobacterium	45.67	0.16%	12	0.05%	7.67	0.02%
Devosia	5100	17.49%	558.67	2.25%	349.67	0.84%
Dyadobacter	317	1.09%	17.33	0.07%	280.33	0.67%
Ensifer	16	0.05%	11	0.04%	1358.67	3.25%
Hydrogenophaga	146	0.50%	11	0.04%	88.67	0.21%
Lysobacter	253.33	0.87%	11.67	0.05%	769.33	1.84%
Massilia	17.33	0.06%	36	0.15%	63	0.15%
Mesorhizobium	2.67	0.01%	6.67	0.03%	13.33	0.03%
Microbacterium	5	0.02%	2	0.01%	14.33	0.03%
Pedobacter	3829	13.13%	5675	22.89%	3374.67	8.06%
Polaromonas	2	0.01%	55.67	0.22%	62.67	0.15%
Pseudomonas	15.67	0.05%	139	0.56%	583.33	1.39%
Shinella	54.67	0.19%	14	0.06%	2.67	0.01%
Sphingomonas	2.67	0.01%	1.67	0.01%	6	0.01%
Sphingopyxis	229	0.79%	56	0.23%	203.33	0.49%
Stenotrophomonas	6441.33	22.09%	6844.67	27.61%	17501.33	41.81%
Taibaiella	650.33	2.23%	20	0.08%	7.33	0.02%
Variovora	8532.33	29.26%	5679	22.91%	2153.33	5.14%
Add up	29158.33	71.32%	24788	45.49%	41858.33	86.76%
Others	11724.33	28.68%	29698.67	54.51%	6390	13.24%
Total	40882.67	100%	54486.67	100%	48248.33	100%

Table S6. Common species of EFBs in *Helvella bachu* associated with different hosts

Host plant	FPE		FPP		FPA		Total
Species	Average abundance	Relative abundance	Average abundance	Relative abundance	Average abundance	Relative abundance	
Variovorax paradoxus	8532.33	29.26%	5679	22.91%	2153.33	5.14%	19.11%
Pedobacter steynii	18	0.06%	5243.67	21.15%	929	2.22%	7.81%
Stenotrophomonas rhizophila	2230	7.65%	3586.67	14.47%	118	0.28%	7.47%
Stenotrophomonas maltophilia	4000.33	13.72%	3218.33	12.98%	17364.67	41.48%	22.73%
Acidovorax radicis	440	1.51%	2927.67	11.81%	8743.67	20.89%	11.40%
Bosea vestrisii	107.67	0.37%	2277.33	9.19%	80.33	0.19%	3.25%
Pedobacter panaciterrae	3571	12.25%	422.33	1.70%	594	1.42%	5.12%
Aminobacter carboxidus	3.33	0.01%	364.33	1.47%	59.67	0.14%	0.54%
Devosia riboflavina	2699	9.26%	350.67	1.41%	67.67	0.16%	3.61%
Devosia oryziradicis	2294.33	7.87%	203.33	0.82%	276	0.66%	3.12%
Pseudomonas silesiensis	15.67	0.05%	139	0.56%	583.33	1.39%	0.67%
Sphingopyxis chilensis	229	0.79%	56	0.23%	203.33	0.49%	0.50%
Polaromonas eurypsychrophila	2	0.01%	55.67	0.22%	62.67	0.15%	0.13%
Bradyrhizobium uaiense	7	0.02%	38.67	0.16%	4	0.01%	0.06%
Stenotrophomonas MUYX sp.	208.33	0.71%	37	0.15%	14.67	0.04%	0.30%
Massilia soli	17.33	0.06%	36	0.15%	63	0.15%	0.12%
Taibaiella JF176697 sp.	650.33	2.23%	20	0.08%	7.33	0.02%	0.78%
Shinella lacus	54.67	0.19%	14	0.06%	2.67	0.01%	0.08%
Agrobacterium radiobacter	7.33	0.03%	14	0.06%	3254.33	7.77%	2.62%
Chryseobacterium aahli sp.	45.67	0.16%	12	0.05%	7.67	0.02%	0.07%
Lysobacter firmicutimachus	253.33	0.87%	11.67	0.05%	769.33	1.84%	0.92%
Hydrogenophaga palleronii	146	0.50%	11	0.04%	88.67	0.21%	0.25%
Ensifer morelensis	16	0.05%	11	0.04%	1358.67	3.25%	1.12%
Dyadobacter psychrophilus	4.33	0.01%	10	0.04%	194.33	0.46%	0.17%
Brevundimonas poindexterae	1520.33	5.21%	7.33	0.03%	9.33	0.02%	1.76%
Acidovorax kalamii	1412.67	4.84%	7.33	0.03%	2867.33	6.85%	3.91%
Dyadobacter endophyticus	312.67	1.07%	7.33	0.03%	86	0.21%	0.44%
Mesorhizobium tamadayense sp.	2.67	0.01%	6.67	0.03%	13.33	0.03%	0.02%
Pedobacter caeni sp.	6.33	0.02%	5.67	0.02%	290.67	0.69%	0.25%
Devosia beringensis	106.67	0.37%	4.67	0.02%	6	0.01%	0.13%
Pedobacter ginsengisoli	233.67	0.80%	3.33	0.01%	1561	3.73%	1.51%
Stenotrophomonas cyclobalanopsidis sp.	2.67	0.01%	2.67	0.01%	4	0.01%	0.01%
Microbacterium oxydans	5	0.02%	2	0.01%	14.33	0.03%	0.02%
Sphingomonas brevis	2.67	0.01%	1.67	0.01%	6	0.01%	0.01%
Add up	29158.33	71.32%	24788	45.49%	41858.33	86.76%	100%
Others	11724.33	28.68%	29698.67	54.51%	6390	13.24%	
Total	40882.67	100%	54486.67	100%	48248.33	100%	

Table S7. The biomarkers of the EFBs of *Helvella bachu* under three host plants.

Group	Species	LDA
FPE	o, Flavobacteriales	4.486258461
	f, Weeksellaceae	4.476454762
	f, Oxalobacteraceae	3.553737492
	g, Chryseobacterium	4.476454762
	s, Pedobacter steynii	4.145447482
	s, Devosia limi	4.068791618
	s, Chryseobacterium vrystaatense	4.473302872
FPP	p, Firmicutes	3.173002048
	c, Gammaproteobacteria	5.007988751
	c, Bacilli	3.171852777
	o, Pseudomonadales	3.352569902
	f, Pseudomonadaceae	3.330400428
	s, Pararhizobium Rhizobium	3.98810496
	g, Pseudomonas	3.330400428
	s, unassigned48 Pseudomonas	3.330400428
FPA	*g, Pseudoflavitalea*	3.167670027
	g, Rhizobium sphaerophysae	3.238198362
	g, Devosia	3.240629719
	s, metagenome 4	3.642880692
	s, uncultured bacterium52	3.167670032

Table S8. Alpha diversity index of EFBs in *Helvella bachu* fruiting bodies associated with various host plants

Sample	Observed feature	Shannon	Simpson	Chao1	ACE	Pielou	Goods coverage
FPE 1	233	2.726	0.88	333.1	334.475	0.347	0.998
FPE 2	237	2.902	0.91	322.583	332.401	0.368	0.998
FPE 3	231	2.87	0.913	332.108	357.128	0.366	0.998
FPP 1	239	1.413	0.486	374.303	359.321	0.179	0.997
FPP 2	221	2.169	0.812	402.138	401.721	0.279	0.997
FPP 3	229	3.012	0.916	381.786	406.003	0.384	0.997
FPA 1	141	1.799	0.688	208.778	271.998	0.252	0.998
FPA 2	209	2.929	0.914	349.87	336.595	0.38	0.998
FPA 3	206	3.163	0.928	397.4	382.099	0.412	0.998

Table S9. ANOSIM intergroup difference analysis of EFBs in *Helvella bachu* fruiting bodies associated with various host plants

Group	Sample num	Group num	P value	R value
Group	9	3	0.005	0.6708
FPE vs FPP	6	2	0.1	0.8519
FPE vs FPA	6	2	0.1	0.7407
FPP vs FPA	6	2	0.1	0.4074

Table S10. OTUs of EFBs in *Helvella bachu* at different developmental stages

**Sample**	**bacteria**	
	**OTU_Num**	**Seqs_Num**
NF1	637	60048
NF2	277	42456
NF3	437	51436
DF1	383	61250
DF2	514	63679
DF3	645	60201
MF1	296	57031
MF2	312	57960
MF3	353	54577
Total	3287	508638

Table S11. EFBs of *Helvella bachu* at different taxonomic levels at different developmental stages

Sample	Species	Genus	Family	Order	Class	Phylum
NF1	289	268	165	98	43	20
NF2	126	119	93	59	23	16
NF3	192	176	115	64	24	16
subtotal	412	368	193	114	47	22
DF1	205	202	134	77	31	18
DF2	257	248	173	103	42	24
DF3	288	282	183	105	47	23
subtotal	471	443	259	156	63	28
MF1	163	159	128	70	31	18
MF2	161	151	111	69	29	19
MF3	162	154	121	69	27	18
subtotal	321	288	184	112	45	24
Total	763	649	336	187	73	30

Table S12. EFBs of *Helvella bachu* at different developmental stages at the phylum level

Phylum	NF	DF	MF
Proteobacteria	75.79%	38.36%	47.37%
Bacteroidota	21.83%	57.76%	51.45%
Firmicutes	1.34%	2.25%	0.53%
Actinobacteriota	0.58%	0.56%	0.25%
Bdellovibrionota	0.03%	0.36%	0.12%
Patescibacteria	0.13%	0.20%	0.06%
unclassified_Bacteria	0.08%	0.07%	0.01%
Spirochaetota	0.01%	0.04%	0.06%
Verrucomicrobiota	0.02%	0.05%	0.03%
Gemmatimonadota	0.04%	0.04%	0.01%
Others	0.14%	0.28%	0.12%
Unassigned	0.00%	0.03%	0.01%
total	100%	100%	100%

Table S13. Shared genus of EFBs in *Helvella bachu* at different developmental stages

Genus	NF averge abundance	DF averge abundance	MF averge abundance
Acidovorax	11.04%	6.00%	6.50%
Rhizobium	0.16%	0.27%	0.66%
Bacteroides	0.12%	0.15%	0.03%
Bosea	0.93%	4.52%	2.68%
Brevundimonas	0.67%	0.29%	0.09%
Caulobacter	0.12%	0.30%	0.09%
Chryseobacterium	2.98%	37.58%	27.91%
Devosia	14.31%	3.73%	0.89%
Legionella	1.68%	2.77%	1.30%
Marinilutecoccus	0.20%	0.35%	0.18%
Pedobacter	15.04%	18.93%	22.49%
Pseudomonas	3.61%	0.07%	0.25%
Ralstonia	0.03%	0.09%	0.02%
Reyranella	0.27%	0.04%	0.01%
Sphingomonas	0.18%	0.17%	0.51%
Sphingopyxis	0.80%	0.49%	0.15%
Stenotrophomonas	15.36%	3.43%	21.10%
Streptococcus	0.04%	0.08%	0.02%
Taibaiella	0.05%	0.03%	0.03%
Variovorax	0.50%	1.63%	4.97%
unclassified_Bacteria	0.08%	0.07%	0.01%
unclassified_Clostridia	0.04%	0.07%	0.03%
unclassified_Clostridia_UCG_014	0.03%	0.02%	0.01%
unclassified_Comamonadaceae	0.85%	0.13%	0.04%
unclassified_Enterobacteriaceae	0.11%	0.31%	0.06%
unclassified_Lachnospiraceae	0.11%	0.15%	0.06%
unclassified_Muribaculaceae	0.15%	0.05%	0.01%
unclassified_Rhizobiaceae	2.76%	1.50%	1.54%
unclassified_Xanthobacteraceae	0.32%	5.06%	1.67%
unclassified_Eubacterium	0.02%	0.02%	0.01%
Others	27.46%	11.70%	6.70%
total	100%	100%	100%

Table S14. EFBs of *Helvella bachu* obtained by culture methods

No.	Sample Name	Latin Name	Similarity	Length
1	EB-1	*Pseudomonas azotoformans*	99.88%	1445bp
2	EB-2	*Pseudomonas fluorescens*	99.88%	1164bp
3	EB-3	*Pseudomonas putida*	99.53%	1439bp
4	EB-4	*Pseudomonas poae*	100.00%	1414bp
5	EB-5	*Pseudomonas poae*	99.88%	1447bp
6	EB-6	*Pseudomonas fluorescens*	99.67%	1163bp
7	EB-7	*Pseudomonas azotoformans*	99.88%	1432bp
8	EB-8	*Pseudomonas gessardii*	99.88%	1433bp
9	EB-9	*Pseudomonas gessardii*	100.00%	1323bp
10	EB-10	*Pseudomonas* sp.	100.00%	952bp
11	EB-11	*Pseudomonas* sp. I11B-02677	100.00%	1128bp
12	EB-12	*Pseudomonas* sp. I11B-02677	99.88%	1128bp
13	EB-13	*Pseudomonas poae*	99.88%	1447bp
14	EB-14	*Stenotrophomonas maltophilia*	85.54%	1450bp
15	EB-15	*Pseudomonas* sp. K94.08	99.63%	1458bp
16	EB-16	*Pseudomonas fluorescens*	99.76%	1164bp
17	EB-17	*Pseudomonas* sp. TK32	99.76%	1445bp
18	EB-18	*Pseudomonas* sp. HH-13	99.75%	995bp
19	EB-19	*Pseudomonas gessardii*	99.88%	1439bp
20	EB-20	*Pseudomonas simiae*	99.38%	1335bp
21	EB-21	*Pseudomonas* sp. FBF110	99.89%	1413bp
22	EB-22	*Serratia* sp. (in: enterobacteria)	99.77%	1442bp
23	EB-23	*Stenotrophomonas maltophilia*	80.02%	1441bp
24	EB-24	*Stenotrophomonas maltophilia*	100.00%	1066bp
25	EB-25	*Pseudomonas* sp. THG-EP13	99.88%	1427bp
26	EB-26	*Pseudomonas azotoformans*	99.88%	1442bp
27	EB-27	*Pseudomonas gessardii*	99.88%	1439bp
28	EB-28	*Pseudomonas* sp. L5B5	99.41%	1462bp
29	EB-29	*Pseudomonas* sp. J3.2C1	99.88%	1433bp
30	EB-30	*Pseudomonas fluorescens*	99.64%	1440bp
31	EB-31	*Pseudomonas* sp.	99.64%	1128bp
32	EB-32	*Pseudomonas* sp. J-4	100.00%	1431bp
33	EB-33	*Pseudomonas* sp. J-4	100.00%	1431bp
34	EB-34	*Pseudomonas* sp.	99.65%	1438bp
35	EB-35	*Pseudomonas fluorescens*	99.88%	1444bp
36	EB-36	*Stenotrophomonas* sp.	97.72%	1438bp
37	EB-37	*Chryseobacterium* sp.	98.08%	1520bp
38	EB-38	*Pseudomonas* sp.	100%	1412bp
39	EB-39	*Microbacterium hibisci*	99%	1433bp
40	EB-40	*Pseudomonas fluorescens*	98%	1459bp
41	EB-41	*Variovorax boronicumulans*	100%	1444bp
42	EB-42	*Chryseobacterium* sp. WG4	97%	1497bp
43	EB-43	*Chryseobacterium* sp.	98%	1456bp
44	EB-44	*Stenotrophomonas rhizophila*	98%	1451bp
45	EB-45	*Pseudomonas fluorescens*	98%	1444bp
46	EB-46	*Microbacterium* sp.	97%	1427bp
